# 3-Ethyl­sulfinyl-2-(4-fluoro­phen­yl)-5,6-methyl­enedi­oxy-1-benzofuran

**DOI:** 10.1107/S160053681001617X

**Published:** 2010-05-08

**Authors:** Hong Dae Choi, Pil Ja Seo, Byeng Wha Son, Uk Lee

**Affiliations:** a Department of Chemistry, Dongeui University, San 24 Kaya-dong Busanjin-gu, Busan 614-714, Republic of Korea; b Department of Chemistry, Pukyong National University, 599-1 Daeyeon 3-dong, Nam-gu, Busan 608-737, Republic of Korea

## Abstract

In the title compound, C_17_H_13_FO_4_S, the 4-fluoro­phenyl ring makes a dihedral angle of 4.92 (4)° with the plane of the 5,6-methyl­enedi­oxy-1-benzofuran fragment. In the crystal, mol­ecules are linked by weak inter­molecular C—H⋯O and C—H⋯F hydrogen bonds.

## Related literature

For the crystal structures of similar 2-aryl-5,6-methyl­enedi­oxy-3-methyl­sulfinyl-1-benzofuran derivatives, see: Choi *et al.* (2007 ▶, 2010 ▶). For the pharmacological activity of benzofuran compounds, see: Aslam *et al.* (2006 ▶); Galal *et al.* (2009 ▶); Khan *et al.* (2005 ▶). For natural products with benzofuran rings, see: Akgul & Anil (2003 ▶); Soekamto *et al.* (2003 ▶).
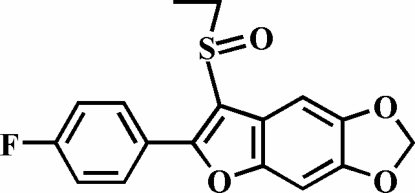


## Experimental

### 

#### Crystal data

C_17_H_13_FO_4_S
*M* *_r_* = 332.33
Triclinic, 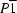

*a* = 7.1081 (9) Å
*b* = 9.631 (1) Å
*c* = 10.708 (1) Å
α = 93.201 (2)°
β = 95.510 (2)°
γ = 105.423 (2)°
*V* = 700.85 (13) Å^3^
*Z* = 2
Mo *K*α radiation
μ = 0.26 mm^−1^
*T* = 173 K
0.40 × 0.36 × 0.28 mm

#### Data collection

Bruker SMART APEXII CCD diffractometer
Absorption correction: multi-scan (*SADABS*; Bruker, 2009 ▶) *T*_min_ = 0.610, *T*_max_ = 0.746
6872 measured reflections
3194 independent reflections
2955 reflections with *I* > 2σ(*I*)
*R*_int_ = 0.021

#### Refinement

*R*[*F* ^2^ > 2σ(*F* ^2^)] = 0.035
*wR*(*F* ^2^) = 0.093
*S* = 1.03
3194 reflections
209 parameters
H-atom parameters constrained
Δρ_max_ = 0.35 e Å^−3^
Δρ_min_ = −0.34 e Å^−3^

### 

Data collection: *APEX2* (Bruker, 2009 ▶); cell refinement: *SAINT* (Bruker, 2009 ▶); data reduction: *SAINT*; program(s) used to solve structure: *SHELXS97* (Sheldrick, 2008 ▶); program(s) used to refine structure: *SHELXL97* (Sheldrick, 2008 ▶); molecular graphics: *ORTEP-3* (Farrugia, 1997 ▶) and *DIAMOND* (Brandenburg, 1998 ▶); software used to prepare material for publication: *SHELXL97*.

## Supplementary Material

Crystal structure: contains datablocks global, I. DOI: 10.1107/S160053681001617X/fl2302sup1.cif

Structure factors: contains datablocks I. DOI: 10.1107/S160053681001617X/fl2302Isup2.hkl

Additional supplementary materials:  crystallographic information; 3D view; checkCIF report

## Figures and Tables

**Table 1:** Hydrogen-bond geometry (Å, °)

*D*—H⋯*A*	*D*—H	H⋯*A*	*D*⋯*A*	*D*—H⋯*A*
C12—H12⋯O4^i^	0.93	2.62	3.380 (2)	140
C16—H16*A*⋯F^ii^	0.97	2.56	3.2090 (17)	125
C17—H17*B*⋯O4^iii^	0.96	2.61	3.469 (2)	149

Symmetry codes: (i) 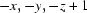; (ii) 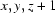; (iii) 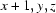.

**Fig. 1.**
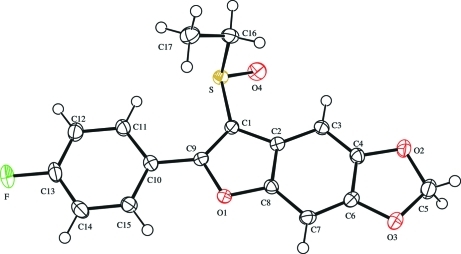
The molecular structure of the title compound with the atom numbering scheme. Displacement ellipsoids are drawn at the 50% probability level. H atoms are presented as a small spheres of arbitrary radius.

**Fig. 2.**
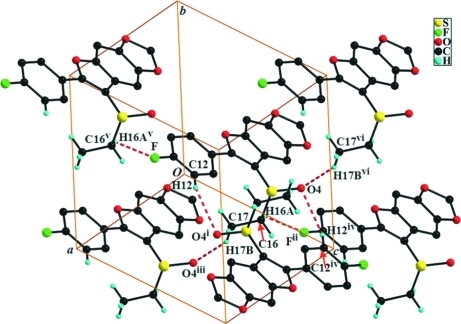
C–H···O and C–H···F interactions (dotted lines) in the crystal structure of the title compound. [Symmetry codes: (i) - x, - y, - z + 1; (ii) x, y, z + 1; (iii) x + 1, y, z; (iv) - x, - y, - z + 1; (v) x, y, z -1 ; (vi) x - 1, y, z.]

## supplementary crystallographic information

### Comment

Compounds involving a benzofuran skeleton show various pharmacological activities such as antifungal (Aslam *et al.*., 2006), antitumor and antiviral (Galal *et al.*., 2009), and antimicrobial (Khan *et al.*., 2005). These compounds occur widely in nature (Akgul & Anil, 2003; Soekamto *et al.*., 2003). As a part of our ongoing studies of the effect of side chain substituents on the solid state structures of 2-aryl-5,6-methylenedioxy-3-methylsulfinyl-1-benzofuran analogues (Choi *et al.*, 2007, 2010), we report the crystal structure of the title compound (Fig. 1). The structure shows both intermolecular C-H···O and C–H···F hydrogen bonds, whereas aromatic π···π interactions were found in 5,6-methylenedioxy-3-methylsulfinyl-2-phenylbenzofuran (Choi *et al.*, 2007) and only intermolecular C–H···O hydrogen bonds were observed in 2-(4-fluorophenyl)-5,6-methylenedioxy-3-methylsulfinyl-1-benzofuran (Choi *et al.*, 2010).

The 5,6-(methylenedioxy)benzofuran unit is essentially planar, with a mean deviation of 0.013 (1) Å from the least-squares plane defined by the twelve constituent atoms. The dihedral angle formed by this plane and the 4-fluorophenyl ring is 4.92 (4)°. The molecular packing (Fig. 2) is stabilized by intermolecular C–H···O hydrogen bonds; the first one between the 4-fluorophenyl H atom and the oxygen of the S═O unit, with a C12–H12···O4^i^, the second one between the methyl H atom of ethyl group and the oxygen of the S═O unit, with a C17–H17B···O4^iii^, respectively (Table 1). The crystal packing (Fig. 2) is further stabilized by intermolecular C–H···F hydrogen bonds between the methylene H atom of ethyl group and the fluorine, with a C16–H16A···F^ii^ (Table 1).

### Experimental

77% 3-Chloroperoxybenzoic acid (202 mg, 0.9 mmol) was added in small portions to a stirred solution of 3-ethylsulfanyl-2-(4-fluorophenyl)-5,6-methylenedioxy-1-benzofuran (253 mg, 0.8 mmol) in dichloromethane (30 mL) at 273 K. After being stirred at room temperature for 4h, the mixture was washed with saturated sodium bicarbonate solution and the organic layer was separated, dried over magnesium sulfate, filtered and concentrated at reduced pressure. The residue was purified by column chromatography (silica gel, ethyl acetate) to afford the title compound as a colorless solid [yield 79%, m.p. 449-450 K; Rf = 0.61 (ethyl acetate)]. Single crystals suitable for X-ray diffraction were prepared by slow evaporation of a solution of the title compound in chloroform at room temperature.

### Refinement

All H atoms were positioned geometrically and refined using a riding model, with C–H = 0.93 Å for aryl, 0.97 Å for methylene, and 0.96 Å for methyl H atoms. *U*_iso_(H) = 1.2*U*_eq_(C) for aryl and methylene H atoms, and 1.5*U*_eq_(C) for methyl H atoms.

### Crystal data

**Table:**

C_17_H_13_FO_4_S	*Z* = 2
*M**_r_* = 332.33	*F*(000) = 344
Triclinic, *P*1	*D*_x_ = 1.575 Mg m^−^^3^
Hall symbol: -P 1	Mo *K*α radiation, λ = 0.71073 Å
*a* = 7.1081 (9) Å	Cell parameters from 4697 reflections
*b* = 9.631 (1) Å	θ = 2.2–27.5°
*c* = 10.708 (1) Å	µ = 0.26 mm^−^^1^
α = 93.201 (2)°	*T* = 173 K
β = 95.510 (2)°	Block, colourless
γ = 105.423 (2)°	0.40 × 0.36 × 0.28 mm
*V* = 700.85 (13) Å^3^	

### Data collection

**Table:**

Bruker SMART APEXII CCD diffractometer	3194 independent reflections
Radiation source: rotating anode	2955 reflections with *I* > 2σ(*I*)
graphite multilayer	*R*_int_ = 0.021
Detector resolution: 10.0 pixels mm^-1^	θ_max_ = 27.5°, θ_min_ = 1.9°
φ and ω scans	*h* = −9→9
Absorption correction: multi-scan (*SADABS*; Bruker, 2009)	*k* = −11→12
*T*_min_ = 0.610, *T*_max_ = 0.746	*l* = −13→13
6872 measured reflections	

### Refinement

**Table:**

Refinement on *F*^2^	Primary atom site location: structure-invariant direct methods
Least-squares matrix: full	Secondary atom site location: difference Fourier map
*R*[*F*^2^ > 2σ(*F*^2^)] = 0.035	Hydrogen site location: difference Fourier map
*wR*(*F*^2^) = 0.093	H-atom parameters constrained
*S* = 1.02	*w* = 1/[σ^2^(*F*_o_^2^) + (0.0462*P*)^2^ + 0.3499*P*] where *P* = (*F*_o_^2^ + 2*F*_c_^2^)/3
3194 reflections	(Δ/σ)_max_ < 0.001
209 parameters	Δρ_max_ = 0.35 e Å^−^^3^
0 restraints	Δρ_min_ = −0.34 e Å^−^^3^

### Special details

**Table:**

Geometry. All esds (except the esd in the dihedral angle between two l.s. planes) are estimated using the full covariance matrix. The cell esds are taken into account individually in the estimation of esds in distances, angles and torsion angles; correlations between esds in cell parameters are only used when they are defined by crystal symmetry. An approximate (isotropic) treatment of cell esds is used for estimating esds involving l.s. planes.
Refinement. Refinement of F^2^ against ALL reflections. The weighted R-factor wR and goodness of fit S are based on F^2^, conventional R-factors R are based on F, with F set to zero for negative F^2^. The threshold expression of F^2^ > 2sigma(F^2^) is used only for calculating R-factors(gt) etc. and is not relevant to the choice of reflections for refinement. R-factors based on F^2^ are statistically about twice as large as those based on F, and R- factors based on ALL data will be even larger.

### Fractional atomic coordinates and isotropic or equivalent isotropic displacement parameters (Å^2^)

**Table:**

	*x*	*y*	*z*	*U*_iso_*/*U*_eq_	
S	0.03741 (5)	0.18237 (3)	0.61999 (3)	0.02096 (11)	
F	0.26284 (17)	0.20685 (12)	0.00053 (8)	0.0408 (3)	
O1	0.28566 (14)	0.56937 (10)	0.50825 (8)	0.0191 (2)	
O2	0.19966 (18)	0.67938 (11)	0.99731 (9)	0.0294 (2)	
O3	0.33831 (17)	0.87862 (11)	0.89214 (10)	0.0291 (2)	
O4	−0.10317 (16)	0.18196 (12)	0.71512 (11)	0.0311 (3)	
C1	0.14785 (19)	0.36484 (14)	0.59545 (12)	0.0173 (3)	
C2	0.18385 (19)	0.48389 (14)	0.69123 (12)	0.0174 (3)	
C3	0.15149 (19)	0.49635 (14)	0.81894 (12)	0.0192 (3)	
H3	0.0949	0.4172	0.8618	0.023*	
C4	0.2106 (2)	0.63511 (15)	0.87430 (12)	0.0204 (3)	
C5	0.2704 (3)	0.83253 (17)	1.00884 (14)	0.0312 (3)	
H5A	0.3770	0.8637	1.0766	0.037*	
H5B	0.1660	0.8749	1.0281	0.037*	
C6	0.2941 (2)	0.75576 (15)	0.81127 (13)	0.0209 (3)	
C7	0.3259 (2)	0.74755 (14)	0.68725 (13)	0.0209 (3)	
H7	0.3804	0.8277	0.6449	0.025*	
C8	0.26789 (19)	0.60610 (14)	0.63121 (12)	0.0176 (3)	
C9	0.21082 (19)	0.42130 (14)	0.48737 (12)	0.0176 (3)	
C10	0.22097 (19)	0.36334 (15)	0.35978 (12)	0.0189 (3)	
C11	0.1684 (2)	0.21477 (15)	0.32548 (13)	0.0234 (3)	
H11	0.1246	0.1505	0.3849	0.028*	
C12	0.1803 (2)	0.16148 (17)	0.20450 (14)	0.0273 (3)	
H12	0.1437	0.0626	0.1817	0.033*	
C13	0.2479 (2)	0.25910 (18)	0.11899 (13)	0.0265 (3)	
C14	0.3012 (2)	0.40604 (17)	0.14766 (13)	0.0266 (3)	
H14	0.3462	0.4690	0.0875	0.032*	
C15	0.2863 (2)	0.45824 (15)	0.26865 (13)	0.0225 (3)	
H15	0.3201	0.5575	0.2896	0.027*	
C16	0.2528 (2)	0.14580 (16)	0.70193 (13)	0.0248 (3)	
H16A	0.3120	0.2226	0.7678	0.030*	
H16B	0.2129	0.0560	0.7414	0.030*	
C17	0.4039 (2)	0.13464 (17)	0.61352 (15)	0.0276 (3)	
H17A	0.3473	0.0563	0.5501	0.041*	
H17B	0.5163	0.1173	0.6604	0.041*	
H17C	0.4435	0.2233	0.5741	0.041*	

### Atomic displacement parameters (Å^2^)

**Table:**

	*U*^11^	*U*^22^	*U*^33^	*U*^12^	*U*^13^	*U*^23^
S	0.02154 (18)	0.01632 (17)	0.02366 (18)	0.00116 (13)	0.00638 (13)	0.00332 (12)
F	0.0605 (7)	0.0474 (6)	0.0187 (4)	0.0201 (5)	0.0118 (4)	−0.0023 (4)
O1	0.0233 (5)	0.0164 (5)	0.0176 (4)	0.0042 (4)	0.0053 (4)	0.0030 (3)
O2	0.0455 (7)	0.0222 (5)	0.0197 (5)	0.0063 (5)	0.0095 (4)	−0.0014 (4)
O3	0.0437 (6)	0.0185 (5)	0.0237 (5)	0.0060 (4)	0.0059 (4)	−0.0024 (4)
O4	0.0287 (6)	0.0290 (6)	0.0365 (6)	0.0040 (4)	0.0162 (5)	0.0079 (5)
C1	0.0169 (6)	0.0165 (6)	0.0184 (6)	0.0038 (5)	0.0036 (5)	0.0018 (5)
C2	0.0157 (6)	0.0171 (6)	0.0194 (6)	0.0043 (5)	0.0028 (5)	0.0021 (5)
C3	0.0202 (6)	0.0189 (6)	0.0186 (6)	0.0039 (5)	0.0046 (5)	0.0036 (5)
C4	0.0212 (6)	0.0232 (7)	0.0178 (6)	0.0072 (5)	0.0043 (5)	0.0017 (5)
C5	0.0412 (9)	0.0237 (7)	0.0253 (7)	0.0030 (6)	0.0080 (6)	−0.0045 (6)
C6	0.0220 (6)	0.0171 (6)	0.0233 (7)	0.0060 (5)	0.0015 (5)	−0.0008 (5)
C7	0.0237 (7)	0.0164 (6)	0.0229 (6)	0.0048 (5)	0.0044 (5)	0.0041 (5)
C8	0.0179 (6)	0.0192 (6)	0.0168 (6)	0.0058 (5)	0.0037 (5)	0.0029 (5)
C9	0.0179 (6)	0.0153 (6)	0.0199 (6)	0.0046 (5)	0.0027 (5)	0.0021 (5)
C10	0.0168 (6)	0.0227 (7)	0.0176 (6)	0.0060 (5)	0.0022 (5)	0.0020 (5)
C11	0.0266 (7)	0.0234 (7)	0.0198 (6)	0.0048 (6)	0.0063 (5)	0.0020 (5)
C12	0.0313 (8)	0.0259 (7)	0.0238 (7)	0.0068 (6)	0.0046 (6)	−0.0028 (6)
C13	0.0290 (7)	0.0376 (8)	0.0158 (6)	0.0139 (6)	0.0048 (5)	−0.0004 (6)
C14	0.0287 (7)	0.0349 (8)	0.0196 (7)	0.0120 (6)	0.0062 (5)	0.0090 (6)
C15	0.0240 (7)	0.0231 (7)	0.0211 (6)	0.0070 (5)	0.0039 (5)	0.0042 (5)
C16	0.0295 (7)	0.0231 (7)	0.0242 (7)	0.0093 (6)	0.0055 (6)	0.0078 (5)
C17	0.0261 (7)	0.0252 (7)	0.0339 (8)	0.0092 (6)	0.0072 (6)	0.0050 (6)

### Geometric parameters (Å, °)

**Table:**

S—O4	1.493 (1)	C7—C8	1.396 (2)
S—C1	1.770 (1)	C7—H7	0.9300
S—C16	1.817 (2)	C9—C10	1.462 (2)
F—C13	1.363 (2)	C10—C11	1.397 (2)
O1—C8	1.371 (2)	C10—C15	1.401 (2)
O1—C9	1.380 (2)	C11—C12	1.385 (2)
O2—C4	1.378 (2)	C11—H11	0.9300
O2—C5	1.420 (2)	C12—C13	1.376 (2)
O3—C6	1.373 (2)	C12—H12	0.9300
O3—C5	1.434 (2)	C13—C14	1.373 (2)
C1—C9	1.370 (2)	C14—C15	1.387 (2)
C1—C2	1.447 (2)	C14—H14	0.9300
C2—C8	1.393 (2)	C15—H15	0.9300
C2—C3	1.412 (2)	C16—C17	1.519 (2)
C3—C4	1.371 (2)	C16—H16A	0.9700
C3—H3	0.9300	C16—H16B	0.9700
C4—C6	1.399 (2)	C17—H17A	0.9600
C5—H5A	0.9700	C17—H17B	0.9600
C5—H5B	0.9700	C17—H17C	0.9600
C6—C7	1.370 (2)		
			
O4—S—C1	107.47 (6)	C1—C9—O1	109.89 (11)
O4—S—C16	106.53 (7)	C1—C9—C10	135.89 (12)
C1—S—C16	97.30 (7)	O1—C9—C10	114.21 (11)
C8—O1—C9	107.08 (10)	C11—C10—C15	118.46 (12)
C4—O2—C5	106.39 (11)	C11—C10—C9	121.86 (12)
C6—O3—C5	105.79 (11)	C15—C10—C9	119.68 (12)
C9—C1—C2	107.48 (11)	C12—C11—C10	121.19 (13)
C9—C1—S	128.49 (10)	C12—C11—H11	119.4
C2—C1—S	124.02 (10)	C10—C11—H11	119.4
C8—C2—C3	120.58 (12)	C13—C12—C11	118.09 (14)
C8—C2—C1	104.70 (11)	C13—C12—H12	121.0
C3—C2—C1	134.72 (12)	C11—C12—H12	121.0
C4—C3—C2	114.24 (12)	F—C13—C14	118.71 (13)
C4—C3—H3	122.9	F—C13—C12	118.20 (14)
C2—C3—H3	122.9	C14—C13—C12	123.09 (13)
C3—C4—O2	127.02 (13)	C13—C14—C15	118.31 (14)
C3—C4—C6	123.81 (13)	C13—C14—H14	120.8
O2—C4—C6	109.17 (12)	C15—C14—H14	120.8
O2—C5—O3	108.58 (11)	C14—C15—C10	120.84 (13)
O2—C5—H5A	110.0	C14—C15—H15	119.6
O3—C5—H5A	110.0	C10—C15—H15	119.6
O2—C5—H5B	110.0	C17—C16—S	111.94 (10)
O3—C5—H5B	110.0	C17—C16—H16A	109.2
H5A—C5—H5B	108.4	S—C16—H16A	109.2
C7—C6—O3	126.72 (13)	C17—C16—H16B	109.2
C7—C6—C4	123.41 (13)	S—C16—H16B	109.2
O3—C6—C4	109.87 (12)	H16A—C16—H16B	107.9
C6—C7—C8	112.81 (12)	C16—C17—H17A	109.5
C6—C7—H7	123.6	C16—C17—H17B	109.5
C8—C7—H7	123.6	H17A—C17—H17B	109.5
O1—C8—C2	110.85 (11)	C16—C17—H17C	109.5
O1—C8—C7	124.01 (12)	H17A—C17—H17C	109.5
C2—C8—C7	125.15 (12)	H17B—C17—H17C	109.5
			
O4—S—C1—C9	−147.47 (12)	C1—C2—C8—O1	−0.34 (14)
C16—S—C1—C9	102.60 (13)	C3—C2—C8—C7	−0.3 (2)
O4—S—C1—C2	31.24 (13)	C1—C2—C8—C7	179.35 (13)
C16—S—C1—C2	−78.69 (12)	C6—C7—C8—O1	−179.57 (12)
C9—C1—C2—C8	0.08 (14)	C6—C7—C8—C2	0.8 (2)
S—C1—C2—C8	−178.86 (10)	C2—C1—C9—O1	0.20 (15)
C9—C1—C2—C3	179.64 (14)	S—C1—C9—O1	179.08 (9)
S—C1—C2—C3	0.7 (2)	C2—C1—C9—C10	178.68 (14)
C8—C2—C3—C4	−0.45 (19)	S—C1—C9—C10	−2.4 (2)
C1—C2—C3—C4	−179.95 (14)	C8—O1—C9—C1	−0.41 (14)
C2—C3—C4—O2	−179.10 (13)	C8—O1—C9—C10	−179.25 (10)
C2—C3—C4—C6	0.7 (2)	C1—C9—C10—C11	−4.3 (2)
C5—O2—C4—C3	−177.56 (14)	O1—C9—C10—C11	174.11 (12)
C5—O2—C4—C6	2.65 (16)	C1—C9—C10—C15	176.13 (15)
C4—O2—C5—O3	−4.39 (17)	O1—C9—C10—C15	−5.45 (17)
C6—O3—C5—O2	4.45 (17)	C15—C10—C11—C12	0.1 (2)
C5—O3—C6—C7	177.68 (14)	C9—C10—C11—C12	−179.47 (13)
C5—O3—C6—C4	−2.82 (16)	C10—C11—C12—C13	0.8 (2)
C3—C4—C6—C7	−0.1 (2)	C11—C12—C13—F	178.92 (13)
O2—C4—C6—C7	179.65 (13)	C11—C12—C13—C14	−0.9 (2)
C3—C4—C6—O3	−179.66 (13)	F—C13—C14—C15	−179.73 (13)
O2—C4—C6—O3	0.13 (16)	C12—C13—C14—C15	0.1 (2)
O3—C6—C7—C8	178.87 (13)	C13—C14—C15—C10	0.9 (2)
C4—C6—C7—C8	−0.6 (2)	C11—C10—C15—C14	−0.9 (2)
C9—O1—C8—C2	0.47 (14)	C9—C10—C15—C14	178.64 (13)
C9—O1—C8—C7	−179.23 (12)	O4—S—C16—C17	176.72 (10)
C3—C2—C8—O1	−179.97 (11)	C1—S—C16—C17	−72.56 (11)

### Hydrogen-bond geometry (Å, °)

**Table:**

*D*—H···*A*	*D*—H	H···*A*	*D*···*A*	*D*—H···*A*
C12—H12···O4^i^	0.93	2.62	3.380 (2)	140
C16—H16A···F^ii^	0.97	2.56	3.2090 (17)	125
C17—H17B···O4^iii^	0.96	2.61	3.469 (2)	149

Symmetry codes: (i) −*x*, −*y*, −*z*+1; (ii) *x*, *y*, *z*+1; (iii) *x*+1, *y*, *z*.
